# Role of abscisic acid in strigolactone-induced salt stress tolerance in arbuscular mycorrhizal *Sesbania cannabina* seedlings

**DOI:** 10.1186/s12870-018-1292-7

**Published:** 2018-05-03

**Authors:** Cheng-Gang Ren, Cun-Cui Kong, Zhi-Hong Xie

**Affiliations:** Key Laboratory of Biology and Utilization of Biological Resources of Coastal Zone, Yantai Institute of Coastal Zone Research, Chinese Academy of Sciences, Yantai, 264003 China

**Keywords:** Strigolactones, Abscisic acid ·arbuscular mycorrhizal, *Sesbania cannabina*, Photosynthesis, Salt stress

## Abstract

**Background:**

Strigolactones (SLs) are considered to be a novel class of phytohormone involved in plant defense responses. Currently, their relationships with other plant hormones, such as abscisic acid (ABA), during responses to salinity stress are largely unknown.

**Results:**

In this study, the relationship between SL and ABA during the induction of H_2_O_2_ – mediated tolerance to salt stress were studied in arbuscular mycorrhizal (AM) *Sesbania cannabina* seedlings. The SL levels increased after ABA treatments and decreased when ABA biosynthesis was inhibited in AM plants. Additionally, the expression levels of SL-biosynthesis genes in AM plants increased following treatments with exogenous ABA and H_2_O_2_. Furthermore, ABA-induced SL production was blocked by a pre-treatment with dimethylthiourea, which scavenges H_2_O_2_. In contrast, ABA production was unaffected by dimethylthiourea. Abscisic acid induced only partial and transient increases in the salt tolerance of TIS108 (a SL synthesis inhibitor) treated AM plants, whereas SL induced considerable and prolonged increases in salt tolerance after a pre-treatment with tungstate.

**Conclusions:**

These results strongly suggest that ABA is regulating the induction of salt tolerance by SL in AM *S. cannabina* seedlings.

**Electronic supplementary material:**

The online version of this article (10.1186/s12870-018-1292-7) contains supplementary material, which is available to authorized users.

## Background

Saline-alkali stress is a serious ecological problem that negatively impacts plant survival, development, and productivity [1]. To survive such stress, plants have established beneficial associations with several microorganisms present in the rhizosphere that can alleviate the stress symptoms [2]. One of the most intensively studied and widespread mutualistic plant–microorganism associations involves arbuscular mycorrhizal (AM) fungi. About 80% of terrestrial plants, including most leguminous species, are able to establish this type of symbiotic relationship with fungi of the division *Glomeromycota* [3]. Indeed, AM symbiosis increases resistance to soil salinity in diverse host plants, such as maize, tomato, and lettuce [4], although the underlying mechanisms are not well characterized [5]. *Sesbania cannabina*, which is a soil-improving legume, is highly adaptable to different adverse climatic conditions, including salinity, drought, and waterlogging stresses. Therefore, developing *S. cannabina–*AM fungi symbiotic relationships might represent a good strategy for improving the fertility of saline soils to enable the growth of agriculturally important crops [6].

Establishing a functioning symbiotic relationship with an AM fungus requires a precise coordination between the partners based on a finely regulated molecular dialogue [7]. The molecular dialogue occurring during the so-called pre-symbiotic stage starts when the host plant produces and exudes strigolactones (SLs) into the rhizosphere. Strigolactones are recognized by AM fungi with an uncharacterized receptor that stimulates hyphal growth and branching, thereby increasing the probability of encountering the host roots [8]. In accordance with their role as signaling molecules in the rhizosphere, SLs are mainly produced in the roots and have been detected in the root extracts of monocotyledonous and dicotyledonous plants [9]. Strigolactones are biosynthetically derived from carotenoids [10] via a sequential oxidative cleavage by two carotenoid cleavage dioxygenases (CCD7 and CCD8), and are apocarotenoids [11], as is abscisic acid (ABA). Once synthesized, SLs are transported acropetally to the shoot or are exuded into the rhizosphere by the ABC transporter PDR1, which was first identified in *Petunia hybrida* [12]. The F-box protein MAX2 and the α/β-fold hydrolase D14/DAD2 are the main candidate components of the SL-perception complex in higher plants [13, 14]. Additionally, MAX2 has diverse roles, including in plant responses to abiotic stress [15]. The importance of SLs during the initial stages of mycorrhizal colonizations is widely recognized. Moreover, emerging evidence indicates that SLs may also affect the subsequent steps of the symbiotic relationship developing in response to environmental stimuli, such as salt and drought stresses [4, 16]. We recently observed that SLs can induce salt stress tolerance in AM *S. cannabina* seedlings that is regulated by the generation of H_2_O_2_ as a signaling molecule [17].

Abscisic acid is an important plant hormone with a critical role in the regulation of salt stress responses [18]. Exposure to stresses, such as salinity, induces the accumulation of ABA, resulting in increased tolerance [19] . Several lines of evidence have revealed that ABA induces the accumulation of H_2_O_2_, which plays an important role in ABA signaling [20] . Moreover, SLs and ABA are critical for the regulation of salt stress responses and the establishment of symbiotic relationships between host plants and AM fungi [4, 21]. Recent studies unveiled the links between ABA and SLs in a number of physiological processes. Exogenous ABA may enhance the accumulation of SLs, especially under stress conditions [22]. Additional studies have demonstrated that SL metabolism and its effects on ABA levels are seemingly opposite in roots and shoots under stress [23]. However, it is unclear whether SL and ABA are involved in a synergistic effect during stress responses. These results suggest the possible relationship between SL and ABA during the induction of plant stress tolerance is complex.

The mechanisms by which SLs enhance plant stress tolerance have so far been largely uncharacterized. Our previous studies confirmed that H_2_O_2_ induces SL production and is critical for SL-induced salt stress tolerance in AM *S. cannabina* seedlings. In the present study, we inoculated *S. cannabina* seedlings with AM fungi and monitored the H_2_O_2_ level, ABA content, and SL accumulation to examine the role of ABA and the relationship between ABA and H_2_O_2_ in the AM fungus-induced increase in SL production in response to salt stress conditions.

## Results

### Effects of ABA levels on strigolactone accumulation

**Fig. 1:**
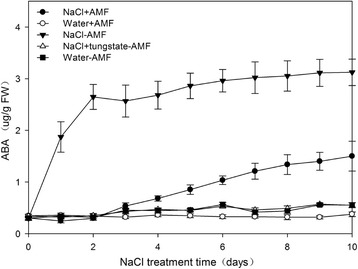
Abscisic acid (ABA) concentration at 1-day intervals in *Sesbania cannabina* seedlings under different salt concentrations with and without an inoculation with an arbuscular mycorrhizal fungus. Values are presented as the mean of three independent experiments. The error bar represents the standard error

In *S. cannabina* seedlings inoculated with an AM fungus, the root colonization rate steadily increased as seedlings grew and differed significantly between sampling times (Table 1). Additionally, the ABA concentration was the highest in salt treated *S. cannabina* seedlings and it was higher in salt-treated AM *S. cannabina* seedlings than in the water-treated controls (Fig. 1), indicating that salt stress may have triggered ABA biosynthesis both in AM and non-AM *S. cannabina* seedlings. Moreover, a bioassay of SL showed that the *P. ramosa* germination rate increased significantly in response to the root extract of salt-stressed AM *S. cannabina* seedlings [17], as did the endogenous SL levels (*m*/*z* 383, *m*/*z*356, and *m/z* 317) (Additional file 1: Table S1).

**Table 1:** Colonization of *Sesbania cannabina* seedling roots by *Funneliformis mosseae*. Values are presented as the mean ± standard error of triplicate samples. Data were analyzed using Duncan’s multiple range test at *p* < 0.05. WAS: weeks after sowing

Time	3 WAS	5 WAS	7 WAS
AMF colonization	8.6 ± 0.54c	26.3 ± 3.1b	45.8 ± 3.73a

**Fig. 2:**
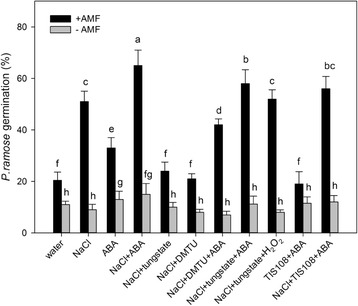
Effect of hydrogen peroxide (H_2_O_2_) inhibitors and abscisic acid (ABA) inhibitors on strigolactone (SL) accumulation in arbuscular mycorrhizal *Sesbania cannabina* seedlings after a 10-day treatment. Germination of *Phelipanche ramosa* seeds induced by *S. cannabina* seedling root extracts. Treatments comprised inhibitors (5 mM DMTU, 5 mM tungstate, and 2 μM TIS108) and 200 mM NaCl, which were applied 1 day before the inoculation with the arbuscular mycorrhizal fungus. Values are presented as the mean of three independent experiments. The error bar represents the standard error. Data were analyzed using Duncan’s multiple range test. Different letters above the error bars indicate statistical significance at *p* < 0.05

To determine whether ABA affects SL generation, we examined the changes in SL levels in AM *S. cannabina* seedlings after ABA and tungstate treatments. As shown in Fig. 4b, ABA did not accumulate in seedlings treated with tungstate, which prevents the formation of ABA from ABA aldehyde by inhibiting ABA-aldehyde oxidase [24]. First, the ABA treatment increased SL levels in AM seedlings but not in non-AM seedlings. Second, the germination bioassay revealed that SL accumulated in salt-stressed AM *S. cannabina* seedlings 10 days after the ABA treatment. However, this ABA-induced increase was not observed in seedlings pre-treated with 5 μM TIS108, a SL synthesis inhibitor (Fig. 2). These results suggested that ABA was important for the AM fungus-induced SL synthesis under salt stress conditions. In contrast, when *S. cannabina* seedlings were treated with an ABA synthesis inhibitor tungstate, the SL content decreased significantly, but the SL level could be recovered by an ABA co-treatment. It also showed that H_2_O_2_ could affect SL accumulation in AM *S. cannabina* seedlings (Fig. 2). Considered together, these results suggested that ABA induced the accumulation of SLs in AM *S. cannabina* seedlings.

### Abscisic acid affects the expression of strigolactone biosynthesis/signaling genes in *S. cannabina* seedlings

**Fig. 3:**
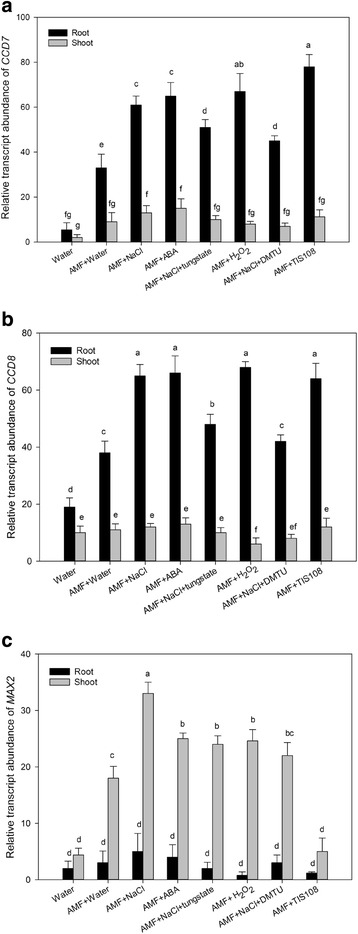
Relative expression levels of SL-biosynthesis/signaling genes in *Sesbania cannabina* seedlings after a 10-day treatment. **a** Relative expression levels of *CCD7*. **b** Relative expression levels of *CCD8*. **c** Relative expression levels of *MAX2*. The expression level of each gene was normalized against that of the *S. cannabina* tubulin gene [41] and is provided relative to the control expression level (= 1). Data are presented as the mean ± standard deviation of three biological replicates. Data were analyzed using Duncan’s multiple range test. Different letters above the error bars indicate statistical significance at *p* < 0.05

To analyze the molecular mechanisms underlying the induction of SL synthesis in AM *S. cannabina* seedlings under salt stress, we examined the effects of ABA and H_2_O_2_ on the expression of *CCD7*, *CCD8*, and *MAX2* homologs in *S. cannabina* seedlings. The expression levels of two SL-biosynthesis genes, *CCD7* and *CCD8*, were up-regulated by NaCl, exogenous ABA, and H_2_O_2_ (Fig. 3). Furthermore, tungstate and DMTU, an H_2_O_2_ scavenger, suppressed the expression of *CCD7* and *CCD8* in salt-stressed AM *S. cannabina* roots. Additionally, seedlings pre-treated with TIS108, which blocks SL biosynthesis, exhibited up-regulated expression of *CCD7* and *CCD8*. We also observed that salt stress, exogenous ABA, and H_2_O_2_ enhanced the expression of the SL-signaling gene, *MAX2*, in AM *S. cannabina* shoots. It is worthy to note that *CCD7* and *CCD8* mainly expressed in root tissue, while *MAX2* in shoot tissue.

### Interactions between ABA and H_2_O_2_ related to strigolactone-induced salt tolerance

**Fig. 4:**
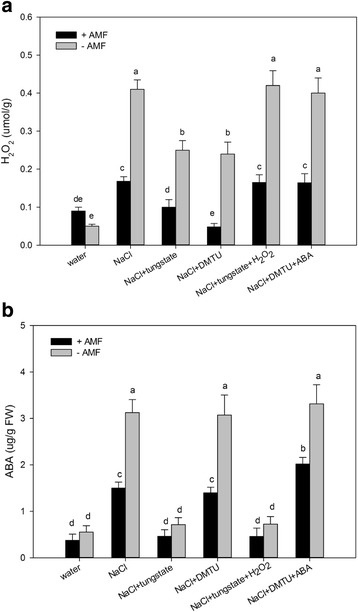
Hydrogen peroxide (H_2_O_2_) concentration and abscisic acid (ABA) accumulation in *Sesbania cannabina* seedlings after 10-day treatments. **a** H_2_O_2_ content. **b** Abscisic acid level. Treatments comprised inhibitors (5 mM DMTU and 5 mM tungstate) and exogenous signaling molecules (10 mM H_2_O_2_ and 100 μM ABA). Values are presented as the mean of three independent experiments. The error bar represents the standard error. Data were analyzed using Duncan’s multiple range test. Different letters above the error bars indicate statistical significance at *p* < 0.05

An earlier study concluded that elevated H_2_O_2_ levels are involved in SL-induced salt stress tolerance [17]. Furthermore, several studies have revealed that ABA can induce the increased production of H_2_O_2_ (Kwak et al. [20]). Therefore, determining whether ABA helps to increase H_2_O_2_ production in AM *S. cannabina* seedlings is warranted. To test this possibility, we pre-treated *S. cannabina* seedlings with tungstate, which prevented the accumulation of ABA and considerably decreased H_2_O_2_ levels in AM *S. cannabina* seedlings (Fig. 4). Additionally, ABA-induced SL production was inhibited by the pre-treatment with DMTU (Fig. 2). These results implied that an increase in ABA content is required for H_2_O_2_ to induce SL production. In contrast, when we pre-treated seedlings with DMTU, we observed no significant effect on the increase in ABA content under salt stress conditions (Fig. 4b). Thus, ABA appears to function upstream of H_2_O_2_ in AM *S. cannabina* seedlings.

### Dependency of ABA biosynthesis for strigolactone-induced salt tolerance

**Fig. 5:**
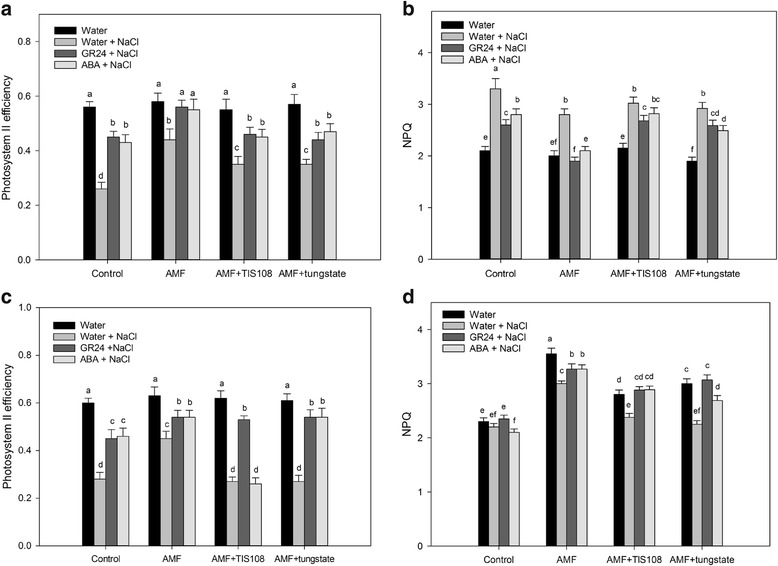
Effects of strigolactone (SL) and abscisic acid (ABA) levels on photosynthetic parameters in salt-stressed *Sesbania cannabina* seedlings. **a** Photosystem II efficiency (ΦPSII) values of *S. cannabina* seedlings after 2 days under salt stress conditions. **b** Non-photochemical quenching of chlorophyll fluorescence (NPQ) of *S. cannabina* seedlings after 2 days under salt stress conditions. **c** Photosystem II efficiency values of *S. cannabina* seedlings after 8 days under salt stress conditions. **d** Non-photochemical quenching of chlorophyll fluorescence of *S. cannabina* seedlings after 8 days under salt stress conditions. Exogenous signaling molecules (1 μM GR24 and 100 μM ABA) were applied 1 day before the *S. cannabina* seedlings were exposed to salt stress conditions. Inhibitors (5 mM tungstate and 2 μM TIS108) were applied 1 day before the exogenous signaling molecules were applied. Values are presented as the mean of six independent experiments. The error bar represents the standard error. Data were analyzed using Duncan’s multiple range test. Different letters above the error bars indicate statistical significance at *p* < 0.05

To study the relationship between ABA- and SL-induced salt tolerances, we analyzed the effects of TIS108, a specific inhibitor of SL biosynthesis, and tungstate, an ABA biosynthesis inhibitor, on SL- and ABA-induced salt tolerances in AM *S. cannabina* seedlings. All plants not treated with salt had similar ΦPSII values (approximately 0.57), while those exposed to 200 mM NaCl had significantly decreased ΦPSII values at 2 and 8 days, especially in seedlings treated with TIS108 and tungstate (Fig. 5a, c). These results suggested that a defect in either ABA or SL accumulation decreased the salt stress tolerance of the seedlings. A pre-treatment with ABA or GR24 (synthetic SL analog) at 2 or 8 days prior to the exposure to salt stress significantly increased ΦPSII values in both control and AM plants. Moreover, GR24 restored the salt tolerance of tungstate-treated plants, while ABA restored the salt tolerance of TIS108-treated plants at 2 days after its application, but was ineffective at 8 days after its application (Fig. 5). Thus, SL was able to rescue the salt stress tolerance of ABA-deficient plants, while ABA could only partially and transiently rescue the defective stress tolerance of the tungstate-treated plants. This observation implied that SL might function downstream of ABA in plant stress responses.

**Fig. 6:**
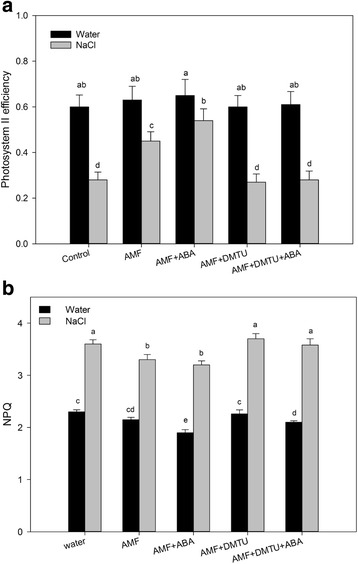
Effect of abscisic acid (ABA) and H_2_O_2_ inhibitors on the photosynthetic parameters of arbuscular mycorrhizal *Sesbania cannabina* seedlings under salt stress conditions. **a** Photosystem II efficiency (ΦPSII) values of *S. cannabina* seedlings after 8 days under salt stress conditions. **b** Non-photochemical quenching of chlorophyll fluorescence (NPQ) of *S. cannabina* seedlings after 8 days under salt stress conditions. Exogenous signaling molecules and inhibitors comprised 100 μM ABA and 5 mM DMTU. Values are presented as the mean of six independent experiments. The error bar represents the standard error. Data were analyzed using Duncan’s multiple range test. Different letters above the error bars indicate statistical significance at *p* < 0.05

Furthermore, ABA-induced salt tolerance was effectively blocked by a pre-treatment with DMTU (Fig. 6), while SL-induced salt tolerance was regulated by DMTU [17]. These results suggested that H_2_O_2_ was also responsible for the observed ABA- and SL-induced salt tolerances.

## Discussion

Plants are sessile in nature. Therefore, to counter biotic and abiotic stresses, such as pathogens, drought, waterlogging, extreme temperatures, salinity, and many others, plants have evolved intricate signaling mechanisms composed of multiple components including plant hormones. Research conducted in the last decade have added SLs to the growing list of plant hormones involved in responses to environmental stresses [25]. In the present study, we revealed that SLs can be regulated by ABA production in *S. cannabina* seedlings (Figs 1, 2). Given the common metabolic precursor shared by ABA and SL, a potential crosstalk between these two hormones was recently proposed, although these hormones appear to be negatively correlated. For example, SL levels reportedly decreased, while ABA content increased in tomato and lettuce plants grown under drought conditions in the absence of AM symbiosis [16]. We also observed that ABA levels increased rapidly under salt stress conditions in plants not colonized by an AM fungus, whereas SL production was very low (Fig. 1). Therefore, this negative correlation might represent a general plant strategy to cope with water-related stresses in the absence of mycorrhization. Conversely, a positive correlation between ABA and SLs has been observed in stress-treated mycorrhizal plants [4, 16]. Similarly, increasing ABA and SL levels were detected in *S. cannabina* seedlings under salt stress conditions in the presence of the AM fungus (Fig. 1)*.* Additionally, ABA enhanced *P. ramosa* germination, whereas tungstate had the opposite effect (Fig. 2). The different relationships between SL and ABA in AM and non-AM *S. cannabina* respectively may indicate that the ABA catabolism was intensively altered by AM inoculation. Overall, it seem that the increase in ABA concentration induced by the AM fungus contributed to the increased accumulation of SLs in salt-stressed *S. cannabina* seedlings.

Our previous results suggested that H_2_O_2_-induced SL production is due to induced/activated NADPH oxidase and is important for SL-induced salt stress tolerance. In this study, we confirmed that ABA has a key role in the production of H_2_O_2_, which regulates the accumulation of SLs. We revealed that H_2_O_2_ production was substantially decreased by a pre-treatment with tungstate (Fig. 4a). Moreover, ABA accumulation was unaffected by a pre-treatment with DMTU (Fig. 4b). Previous investigations also concluded that ABA causes H_2_O_2_ to accumulate in the apoplast, which is dependent on NADPH oxidase and is important for ABA signaling [20, 26]. These results suggest that ABA may act upstream of H_2_O_2_ in the signaling pathway of AM *S. cannabina* seedlings. Furthermore, we provided evidence that H_2_O_2_ is important for ABA-induced SL production. We also observed that SL production was considerably decreased by a pre-treatment with tungstate or DMTU (Fig. 2). An ABA treatment partly restored the *P. ramosa* germination rate in DMTU-treated mycorrhizal plantlets. Simultaneously, an H_2_O_2_ treatment significantly restored the *P. ramosa* germination rate of tungstate-treated mycorrhizal plantlets (Fig. 2). Taken together, these results implied that ABA induced SL accumulation in AM *S. cannabina* seedlings, which may be partially dependent on increased H_2_O_2_ levels.

In this study, we confirmed that ABA is involved in the SL-induced salt tolerance of *S. cannabina* seedlings inoculated with an AM fungus. Exposure to tungstate decreased SL levels and ΦPSII values in salt-stressed AM *S. cannabina* seedlings (Figs 2, 5a, c). Furthermore, an ABA treatment significantly restored the *P. ramosa* germination rate in TIS108-treated mycorrhizal plantlets, indicating that the ABA-induced salt stress tolerance of AM *S. cannabina* seedlings required the accumulation of SL (Fig. 2). The relationship between SL and ABA during plant responses to abiotic stresses has been rarely studied. Many investigations have considered ABA as the ‘abiotic stress hormone’ because its biosynthesis is rapidly induced by exposures to environmental stresses, especially water-related stresses such as drought and salinity [27–29]. The reported increase in the ABA content of AM plants subjected to abiotic stress likely contributes to the enhanced tolerance of these plants to environmental stresses [4, 30]. Additionally, increased SL levels were detected in lettuce plants grown in the presence of the AM fungus *Rhizophagus irregularis* under salt stress conditions [4]. A similar trend has been observed in drought-treated lettuce and tomato plants [16]. These observations combined with our results indicate that SL-induced salt stress tolerance of AM plants may be mediated by a complex set of signal transduction pathways with ABA as a common signaling molecule.

## Conclusions

In summary, we uncovered a dynamic interplay between SLs, ABA, and H_2_O_2_ produced in response to an AM fungus that contributed to the salt stress tolerance of *S. cannabina* seedlings. Following the perception of the ABA signal, H_2_O_2_ was rapidly produced, which then increased the accumulation of SLs, and ultimately enhanced the salt stress tolerance of the seedlings. Additional studies are needed to provide the genetic evidence of the involvement of ABA in H_2_O_2_-induced SL generation and to identify the critical signaling components between SL production and salt stress responses in *S. cannabina* seedlings inoculated with an AM fungus. Such studies will help to elucidate the molecular mechanism underlying the ABA- and SL-induced salt tolerance of AM plants.

## Methods

### Plant materials and treatments

*Sesbania cannabina* (Retz.) Pers. seeds were obtained from the Shandong Academy of Agricultural Sciences, Shandong, China. Before sowing, the seeds were surface-sterilized in 5% sodium hypochlorite for 5 min and rinsed several times with distilled water. The seeds were germinated at 28 °C in distilled water and then sown in trays containing autoclaved zonolite. After 2 weeks, individual seedlings were transferred to 1-L pots containing autoclaved zonolite inoculated with 10 g inoculum (approximately 121 spores). The original inoculum [AM fungus *Funneliformis mosseae*] was propagated in pot-cultured *Trifolium repens* plants for 8 weeks, and included infected roots, hyphae, spores, and substrates. The growth conditions were as follows: 12 h photoperiod, 25/17 °C (day/night), and light intensity of 600 μmol m^− 2^ s^− 1^. Three-week-old seedlings were used for all treatments with 200 mM NaCl solutions. The reagents used as specific scavengers or inhibitors [5 mM DMTU (H_2_O_2_ scavenger) [31], 5 mM sodium tungstate [32], and 2 μM TIS108 (the most potent and specific SL biosynthesis inhibitor) [33]] were purchased from Sigma-Aldrich (St Louis, MO, USA). Additionally, 10 mM H_2_O_2_, 100 μM ABA, and 1 μM GR24 (synthetic SL analog) were used as exogenous signaling molecules [34]. Plantlet leaves were sprayed with 100 μL exogenous signaling molecule or inhibitor solution. Treatments were completely randomized and replicated three times. An equal volume of distilled water was applied as the control treatment. Unless stated otherwise, inhibitors were applied 24 h before the exogenous signaling molecules were applied.

### Photosynthetic parameters

Gas exchange and modulated chlorophyll fluorescence parameters were simultaneously detected using a LI-6400XTR Portable Photosynthesis System (Li-Cor, Lincoln, NE, USA) equipped with a 6400–40 Leaf Chamber Fluorometer (Li-Cor). The leaves were incubated in darkness for 20 min before being analyzed. The minimal fluorescence level of the dark-adapted leaves was measured using a modulated pulse (< 0.05 μmol m^− 2^ s^− 1^ for 1.8 s), while the maximal fluorescence level was measured after applying a saturating actinic light pulse of 8000 μmol m^− 2^ s^− 1^ for 0.7 s. The actinic light intensity was increased to 1000 μmol m^− 2^ s^− 1^ and then maintained for about 30 min. The steady-state fluorescence yield was also recorded. A saturating actinic light pulse of 8000 μmol m^− 2^ s^− 1^ for 0.7 s was then applied to induce the maximum fluorescence yield by temporarily inhibiting photosystem II (PSII) photochemical activities. The minimum steady-state fluorescence yield was determined during a brief interruption of the actinic light irradiation in the presence of far-red light (λ = 740 nm). Finally, PSII efficiency (ΦPSII) and non-photochemical quenching of chlorophyll fluorescence were calculated according to the method described by Maxwell and Johnson [35].

### Measurement of arbuscular mycorrhizal fungal colonization

The percentage of roots colonized by mycorrhizal fungi was calculated using the gridline intersection method [36] after samples were stained with trypan blue [37].

### Measurement of H_2_O_2_

Seedlings were harvested 10 days after treatments to measure the H_2_O_2_ content. The H_2_O_2_ concentration was determined by monitoring the absorbance of titanium peroxide at 415 nm following the method of Brennan and Frenkel [38]. One unit of H_2_O_2_ was defined as the chemiluminescence produced by the internal standard of 1 μM H_2_O_2_ g^− 1^ fresh weight.

### Extraction of ABA and HPLC analysis

Seedlings collected for the ABA analysis were immediately frozen with liquid N_2_ and stored at − 80 °C. Root tissue (0.5 g) was ground in a mortar and homogenized in 5 ml pre-cooled 80% aqueous acetone (4:1, *v*/v) supplemented with 10 mg/l butylated hydroxytoluene. Extracts were eluted through a Sep Pak C18-cartridge (PerkinElmer, Waltham, MA, USA) to remove polar compounds and then purified using ethyl acetate and NaHCO_3_ as described by Sweetser and Vatvars [39]. After centrifuging samples at 14,000×g for 30 min at 4 °C, the supernatant was acidified to pH 3.0 with H_2_SO_4_ and then treated three times with an equal volume of ethyl acetate. The extract phase was evaporated at 40 °C using the RE-100 rotary evaporator (Bibby Sterlin Ltd., Stone Staffordshire, UK). The dried samples were re-dissolved in chromatography-grade acetonitrile with 0.1 M acetic acid and then analyzed by HPLC using a Hypersil-ODS C18 column (4.6 mm × 250 mm, 5 μm). The mobile phase comprised acetonitrile: 0.03 M acetic acid (30:70, v/v) at a flow rate of 1 ml/min. Abscisic acid was detected spectrophotometrically at 254 nm at 25 °C. The retention time was 13.37 min. Abscisic acid standard curves (20–3000 ng/ml) were used to quantify ABA contents (R^2^ = 0.991).

### Extraction of strigolactones and quantification by a germination bioassay

Root tissue (0.5 g) was collected and ground in a mortar with liquid nitrogen and then treated with 1 ml ethyl acetate in a 3-ml glass tube. The tubes were vortexed and sonicated for 10 min in a Branson 3510 ultrasonic bath (Branson Ultrasonics, Danbury, CT, USA). Samples were then centrifuged at 4000×g for 5 min in an MSE Mistral 2000 centrifuge (Mistral Instruments, Leicester, UK). The organic phase was carefully transferred to 1-ml glass vials and stored at − 20 °C until used in the germination bioassays.

Strigolactones were identified by liquid chromatography–tandem mass spectrometry using a Quattro LC mass spectrometer (Micromass, Manchester, UK) equipped with an electrospray source as previously described (Kong et al. [17]).

The germination bioassays with *Phelipanche ramosa* seeds were conducted according to the method developed by Yoneyama [40]. Approximately 20 surface-sterilized *P. ramosa* seeds were placed on 6-mm glass fiber discs (Whatman) and about 90 discs were incubated in a Petri dish (9 cm diameter) lined with filter paper moistened with 6 ml sterile Milli-Q water. The seeds required a 12-day pre-conditioning incubation at 21 °C in darkness before they became responsive to the germination stimulants. A sterile Petri dish (5 cm diameter) was lined with filter paper moistened with 50 μl *S. cannabina* root extract, which was allowed to evaporate before the pre-conditioned seeds were added. The seeds were then treated with 650 μl sterile Milli-Q water. The synthetic germination stimulant GR24 (10^− 6^ M) and demineralized water were included as positive and negative controls in each bioassay. The Petri dishes were sealed, enclosed in polyethylene bags, and incubated at 25 °C in darkness for 7 days. The germinated and non-germinated seeds were counted using a stereoscope. Seeds were considered germinated if the radicle protruded through the seed coat.

### Quantitative real-time PCR

Root samples from three seedlings were pooled to represent a single biological replicate. Total RNA was extracted from the pooled root samples using TRIzol (Invitrogen, Carlsbad, CA, USA) following the manufacturer’s instructions. Three biological replicates were prepared. The extracted RNA was used as the template for reverse transcription reactions completed with the GoScript™ Reverse Transcription System (Promega, Madison, WI, USA). Gene-specific primers were designed using the Primer Premier program (version 5.0) (Premier Biosoft International). The quantitative real-time (qRT)-PCR analysis was conducted with an iCycler iQ Real-time PCR Detection System (Bio-Rad) with three technical replicates. Each reaction was completed in a total volume of 20 μl, which included 0.2 μM primer pairs, 2 μl diluted cDNA, and 10 μl 2× SYBR Green PCR Master Mix (TaKaRa Bio Inc., Dalian, China). The PCR program was as follows: 95 °C for 30 s; 40 cycles of 95 °C for 5 s, 58 °C for 15 s, and 72 °C for 20 s. Tubulin genes were used as the internal controls and relative gene expression levels were calculated using the 2^−ΔΔCt^ method [41]. The sequences of the qRT-PCR primers are listed in Table 2.

**Table 2:** Sequences of qRT-PCR primers used to analyze strigolactone-related genes

Gene	GenBank accession no.	Primers
*ScCCD7*	MF683952	5’-TGCTCCATCCACAACAGA-3′ 5’-CTCCATAAGGCCAACACC-3’
*ScCCD8*	MF683953	5’-TGGTCTAGCCACAAAGAA-3′ 5’-ATGAACATGGGAAAGGAT-3’
*ScMAX2*	MF683954	5′-TTGGATTAGGGTTGGTGA-3′ 5’-CTGAAGGTGCCGTGAGTA-3’

The nucleotide sequence reported in this table has been submitted to GenBank with accession numbers MF683952, MF683953 and MF683954

### Statistical analysis

All data were analyzed using Microsoft Excel (Redmond, WA, USA) and the values for each treatment are presented as the mean ± standard deviation of three replicates. A one-way analysis of variance was completed with the SPSS Statistics 17.0 software (SPSS, Inc., Chicago, IL, USA). Duncan’s multiple range test was used to compare mean values at the *p* < 0.05 significance level.

## Additional file

Additional file 1: **Table S1** Effect of different treatments on the relative abundances of three SLs candidates m/z 383, m/z 356 and m/z 317 in *S. cannabina* seedlings extract.Data were analyzed using Duncan’s multiple range test. Different letters indicate statistical significance at *p* < 0.05. (XLSX 13 kb)

## Abbreviations

ABA: Abscisic acid
AM: Arbuscular mycorrhizal
CCD: Carotenoid cleavage dioxygenases
DMTU: Dimethylthiourea
*m/z*: Mass to charge ratio
MAX2: More axillary growth2
NPQ: Non-photochemical quenching of chlorophyll fluorescence
SL: Strigolactone
TIS108: 6-phenoxy-1-phenyl-2-(1H-1,2,4-triazol-1-yl) hexan-1-one
ΦPSII: Photosystem II efficiency
